# Cost-Effectiveness of Alternative Blood-Screening Strategies for West Nile Virus in the United States

**DOI:** 10.1371/journal.pmed.0030021

**Published:** 2006-01-24

**Authors:** Caroline T Korves, Sue J Goldie, Megan B Murray

**Affiliations:** **1**Department of Epidemiology, Harvard School of Public Health, Boston, Massachusetts, United States of America; **2**The Earth Institute, Columbia University, New York, New York, United States of America; **3**The Harvard Center for Risk Analysis, Department of Health Policy and Management, Harvard School of Public Health, Boston, Massachusetts, United States of America; **4**Infectious Disease Unit, Massachusetts General Hospital, Boston, Massachusetts, United States of America; New York City Department of Health and Mental HygieneUnited States of America

## Abstract

**Background:**

West Nile virus (WNV) is endemic in the US, varying seasonally and by geographic region. WNV can be transmitted by blood transfusion, and mandatory screening of blood for WNV was recently introduced throughout the US. Guidelines for selecting cost-effective strategies for screening blood for WNV do not exist.

**Methods and Findings:**

We conducted a cost-effectiveness analysis for screening blood for WNV using a computer-based mathematical model, and using data from prospective studies, retrospective studies, and published literature. For three geographic areas with varying WNV-transmission intensity and length of transmission season, the model was used to estimate lifetime costs, quality-adjusted life expectancy, and incremental cost-effectiveness ratios associated with alternative screening strategies in a target population of blood-transfusion recipients. We compared the status quo (baseline screening using a donor questionnaire) to several strategies which differed by nucleic acid testing of either pooled or individual samples, universal versus targeted screening of donations designated for immunocompromised patients, and seasonal versus year-long screening. In low-transmission areas with short WNV seasons, screening by questionnaire alone was the most cost-effective strategy. In areas with high levels of WNV transmission, seasonal screening of individual samples and restricting screening to blood donations designated for immunocompromised recipients was the most cost-effective strategy. Seasonal screening of the entire recipient pool added minimal clinical benefit, with incremental cost-effectiveness ratios exceeding US$1.7 million per quality-adjusted life-year gained. Year-round screening offered no additional benefit compared to seasonal screening in any of the transmission settings.

**Conclusions:**

In areas with high levels of WNV transmission, seasonal screening of individual samples and restricting screening to blood donations designated for immunocompromised recipients is cost saving. In areas with low levels of infection, a status-quo strategy using a standard questionnaire is cost-effective.

## Abstract 2

Screening all blood donations in all States to avoid transmission to blood transfusion recipients is not cost-effective.

## Introduction

The first US-based case of West Nile virus (WNV) disease was reported in New York in 1999 [1]; since then, the virus has spread across the country, leading to 16,637 detected cases of WNV-associated illness and 647 WNV-associated deaths from 1999 to 2004, inclusive (http://www.cdc.gov/).

WNV is a neuropathic flavivirus, transmitted from birds to humans via the mosquito vector. Naturally acquired infection is most often asymptomatic; about 20% of those infected develop a flu-like illness characterized by fever, while a smaller proportion (less than 1%) develop neuroinvasive disease (NI). WNV-associated NI can result in death or in serious sequelae; these poor outcomes occur most commonly in elderly and immunocompromised patients [2].

The Centers for Disease Control and Prevention (CDC) recently reported on the first 23 patients infected through blood transfusion, six of whom died [3]. Although they are a very small minority of the WNV cases reported, transfusion-acquired infections are potentially avoidable, and public health authorities moved quickly to try to safeguard the blood supply from the virus. The Food and Drug Administration (FDA) accelerated the approval of two investigational nucleic acid-based blood-safety tests, and screening of donations was initiated by July 2003 [4]. WNV was subsequently detected in 818 blood donations [3], leading public health officials to conclude that the screening program had prevented at least some WNV transmission. Nonetheless, at least six cases of transfusion-associated WNV have been reported since the initiation of the testing, raising concern that current testing strategies may be inadequate.

The choice of optimal screening strategy will likely depend on the prevalence of WNV infection among donors, the duration of the epidemic season, the dilution of the pooled samples, and the underlying health status of blood-transfusion recipients. Although screening of blood by nucleic acid test (NAT) is currently mandated by the FDA, actual implementation of screening strategies is largely at the discretion of the individual states and the blood-collection agencies. For example, the FDA specifies that testing must be performed in all states from May until the end of October, but allows year-long testing if states deem it necessary [4]. Similarly, NAT can be performed on individual blood donations or on pooled samples. Compared to individual sample testing, the pooling of samples lowers screening costs at the expense of diminished assay sensitivity [5]. Furthermore, current policy mandates screening blood for all transfusion recipients. A subset of transfusion recipients may have an impaired immune response owing to malignancy, HIV, or to the use of immunosuppressive drugs. Targeted screening of blood designated for immunocompromised patients, who are most at risk of developing severe consequences following WNV infection, may be an alternative to universal screening. This approach has been used to prevent transfusion-associated cytomegalovirus infection in immunocompromised patients [6].

To identify the most medically effective and cost-effective screening strategies under a range of different circumstances, we conducted a cost-effectiveness analysis of alternative strategies for WNV blood screening and considered the effects of variable assay characteristics, transfusion outcomes, and pricing that may affect current and future policy decisions.

## Methods

### Analytic Overview

We developed a state-transition model to simulate the natural history of WNV infection transmitted from blood donors to transfusion recipients. The model was used to estimate lifetime costs, life expectancy, and quality-adjusted life expectancy for different blood-screening strategies in a target population of people receiving blood transfusions. Since the cost-effectiveness of screening depends on the prevalence of infectious cases among blood donors, the analyses were performed for three different populations of donors residing in states in which the incidence of WNV varies in intensity and duration of the natural transmission season. Parameters describing the natural history of transfusion-associated WNV were derived from the literature. The implications of alternative parameter values were evaluated in sensitivity analyses. We chose the 95% confidence interval of estimated probabilities to establish clinical parameter ranges, and we consulted experts in the field to establish price and assay ranges.

We adopted a societal perspective and followed the reference-case recommendations of the Panel on Cost-Effectiveness in Health and Medicine [8]. Future costs and QALYs saved were discounted at an annual rate of 3%. Alternative screening strategies were compared using the ICER*.* We assessed the internal consistency and face validity of our model by predicting the number of transfusion cases for each state for 2002 and comparing these outputs with the number of cases reported to state health departments for that year (R. J. Powell, K. Signs, R. Timperi, K. A. Winpisinger, personal communications). We conducted extensive sensitivity analyses to evaluate the stability of our conclusions over a wide range of parameter estimates and assumptions.

### Strategies

We considered four main WNV-screening strategies to be added to baseline screening of blood donors for general infectious diseases. Baseline screening involves the distribution of a donor questionnaire that elicits information about any recent history of fever and is designed to ensure that the blood of individuals with active infections is excluded from the blood supply. Blood centers design their own questionnaires about general health and well being based on donor-eligibility rules established by the FDA (http://americanredcrossblood.org/). An abbreviated physical examination includes checking for abnormalities in blood pressure, pulse, and temperature (http://www.aabb.org/). The four WNV-screening strategies to be superimposed on the questionnaire include: (1) nucleic acid testing of minipools of 16 samples (MP16-NAT) followed by testing the samples in a reactive pool by individual nucleic acid test (ID-NAT); (2) nucleic acid testing of minipools of six samples (MP6-NAT) followed by individual testing of the samples in a reactive pool by ID-NAT; (3) nucleic acid testing of individual samples by ID-NAT; and (4) individual nucleic acid testing on blood donations designated for immunocompromised recipients only. We also evaluated the implications of “seasonal targeting,” meaning that supplemental strategies would be brought into operation only during the “high incidence” WNV season from May through to the end of October in contrast to year-round screening.

### Model Structure and Assumptions

**Figure 1:**
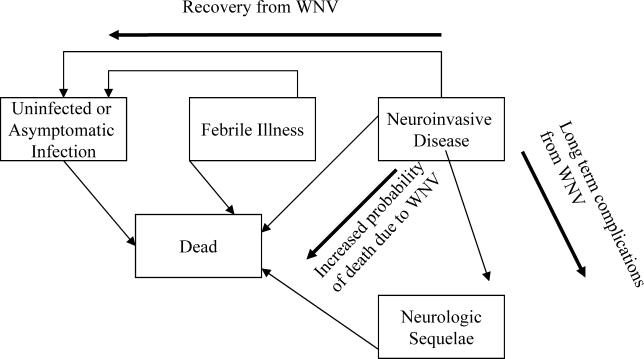
Post-Transfusion Health States Five post-transfusion health states were identified. Following transfusion, individuals entered the uninfected or asymptomatic infection state, febrile-illness state, or NI state and progressed to other health states in the direction of the arrows. Individuals were followed until death.

We constructed a Markov model for each of the three WNV-transmission scenarios described in Table 1. Markov models depict the natural history of disease as an evolving sequence of health states, defined to capture important clinical outcomes, each of which is associated with specific costs. The time horizon of the analysis incorporates a transfusion recipient's lifetime and is divided into equal weekly increments during which transitions between health states occur. The following five mutually exclusive health states included in this model describe the various WNV infection and disease statuses of people after they have received a blood transfusion: (1) no WNV infection or asymptomatic infection; (2) WNV infection leading to febrile illness only; (3) WNV infection leading to NI with no long-term sequelae; (4) WNV infection leading to NI associated with long-term sequelae leading to either institutionalized or home care; or (5) death (Figure 1). Individuals entering the model are assumed to be uninfected at the time of transfusion and are assigned gender, age, age-specific post-transfusion life expectancy, and immune status based on the distribution of these characteristics in previously studied transfused populations [9].

**Table 1:**
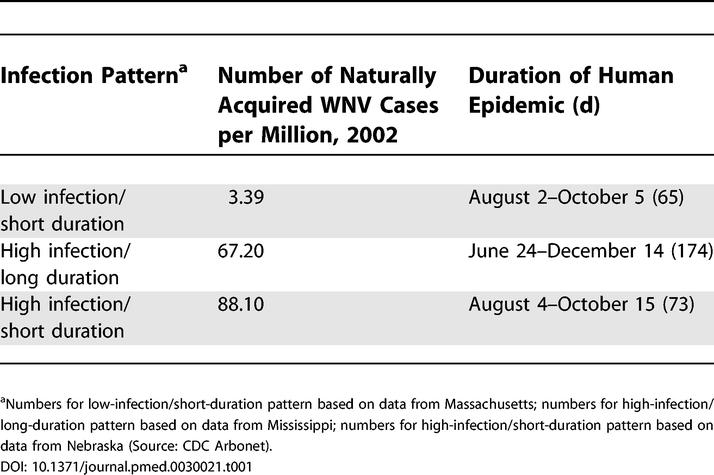
WNV Disease Characteristics in General Population Giving Rise to Risk in Hypothetical Transfusion Cohorts

Transition probabilities derived from a review of the literature were used to move transfusion recipients through different health states over time until all the members of the transfused cohort had died. For example, upon transfusion, each individual faced a risk of transfusion-acquired WNV infection. The risk of infection varied depending on the sensitivity and specificity of the specific screening strategies being analyzed. We assumed that a WNV-positive blood donation always resulted in infection but that the risk of disease in those who were infected was higher in elderly and immunocompromised patients. Once infected, individuals progressed through a week-long incubation period after which they could either remain asymptomatic or make the transition to a health state characterized by febrile illness only or by NI, with or without long-term sequelae or death. Patients who moved into the febrile-illness-only state either recovered or died within 1 wk from causes unrelated to WNV infection, while those in the NI state faced a weekly probability of recovery, death from WNV, or death from unrelated causes.

### Natural History of Transfusion-Acquired WNV

We estimated the weekly probability that a unit of transfused blood would be infected with WNV in each of the three transmission scenarios using a method developed by Biggerstaff et al. [10,11]. Following this approach, we used data on the dates of onset of detected neuroinvasive cases, estimated distributions of WNV incubation, symptomatic and viremic periods, and the ratio of detected to undetected infections to estimate the number of potential blood donors with WN viremia at each time point over a 1-y period. Dates of onset of naturally acquired WNV-associated NI for each of the three scenarios, which were based on corresponding WNV-associated NI case data, were obtained from the CDC Arbonet surveillance Team for 2002. Based on data from previous serological studies, we estimated that, for every reported case of WNV-associated NI, 140 cases of WNV infection had occurred that were either asymptomatic or had presented with fever only [12]. We then used the dates of symptom onset of the detected cases as “anchor times” from which we generated, for each non-NI case, a date of infection, onset of viremia, onset of symptoms, resolution of symptoms, and resolution of viremia. By dividing the number of viremic individuals by the state population, we determined the probability of WNV infection among the general population for a given week for each scenario.

**Figure 2:**
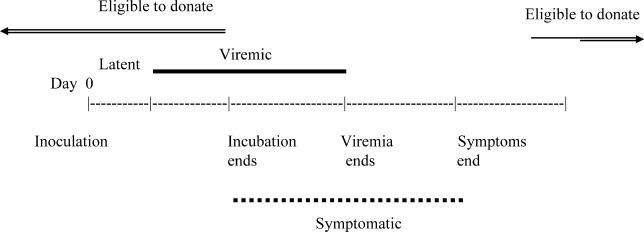
Potential Donor Time for an Infected Individual A potential blood donor who develops WNV symptoms is viremic and eligible to donate for a longer period of time as the latent period decreases and the incubation period increases. A potential blood donor who recovers from symptoms in a short amount of time may be eligible to donate before viremia ends.

Figure 2 illustrates the potential viremic donor times. Without detection by NAT, asymptomatic individuals could donate on any day. Durations of the incubation, viremic, and symptomatic periods were derived from the literature; the ranges of these values are given in Table 2. Once the time line of infection was generated for each case, we counted the number of potential donors who were viremic on each day during the course of a year, and generated a curve representing the prevalence of viremic blood donors over the time course of the simulation. We repeated the simulation 1,000 times to obtain Monte Carlo averages of the prevalence of viremic donors, and these averages were then used to estimate the probability that a blood-transfusion recipient would be transfused with WNV-infected blood. By designing our model to output the weekly probability of a viremic donation, we were able to evaluate the impact of implementing supplemental assay screening for selected weeks within the year.

**Table 2:**
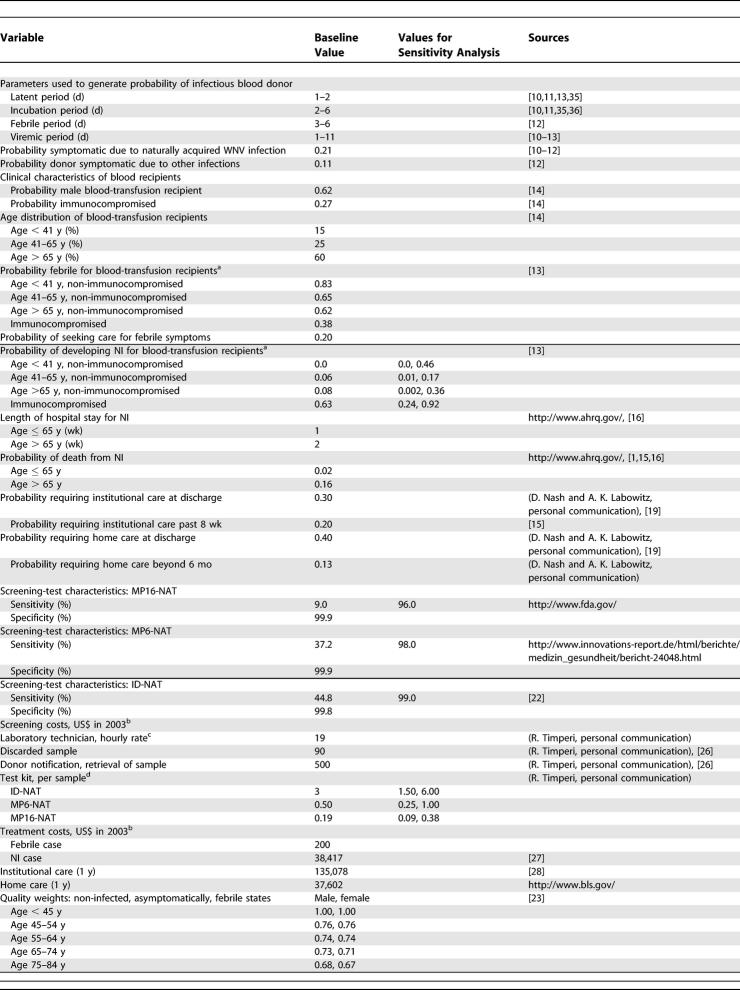
Model Variables: Baseline Values and Values Used in Sensitivity Analysis

**Table 2:**
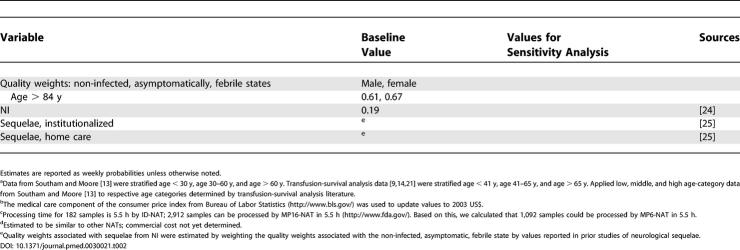
Continued

### Clinical Data

Clinical parameters [13–20] used in the model are shown in Table 2. Since there are few data on death from NI due to WNV, we used national hospital data on death from NI due to multiple causes collected by the Healthcare Cost and Utilization Project (http://www.ahrq.gov/hcupnet/). We utilized unpublished follow-up data on 36 hospitalized patients with WNV to estimate long-term recovery from WNV-associated NI (D. Nash and A. K. Labowitz, personal communication). Distributions of age, sex, and immune status for transfusion recipients were based on transfusion look-back studies [14]. Since there are no population-based studies that reported the probability of developing febrile illness or NI after acquiring transfusion-associated WNV infection, we relied on data from an experimental study of deliberate WNV inoculation of humans with cancer [13]. Age and sex-specific background mortality for the blood recipients was based on a transfusion-cohort study [21].

### Screening Tests

Table 2 summarizes baseline estimates of test sensitivity and specificity for ID-NAT [22]. ID-NAT sensitivity was derived from a study [22] which compared methods of virus detection in macaques experimentally inoculated with WNV. The sensitivities of MP6-NAT and MP16-NAT relative to ID-NAT were estimated from data on the proportion of ID-NAT–positive samples that were identified by minipool tests; FDA meeting transcripts (http://www.fda.gov/ohrms/dockets/ac/03/transcripts/4014T1.htm) and a publicly available Web site (http://www.innovations-report.de/html/berichte/medizin_gesundheit/bericht-24048.html) provided these data. In the absence of definitive data, we assumed that specificity of these tests was high.

### Health-Related Quality of Life

We used a quality-of-life well-being index to assign quality weights to uninfected and asymptomatically infected individuals based on their age and sex [23]. Quality weights for the NI state were based on a study of herpes simplex infection of the central nervous system [24]. The quality weights for neurological sequelae states requiring institutionalized care and home care were based on a study of neurological sequelae resulting from *Haemophilus influenzae* vaccination [25].

### Costs

The cost estimates [26–28] used in the base case are shown in Table 2. Direct costs for screening blood donors using WNV assays included screening kit and reagents, laboratory technician fees, and the costs of a discarded false positive, donor notification, and retrieval of a test-positive sample. Since WNV assays have not yet been priced, we estimated their cost from studies of similar assays for other viruses. Other screening costs were obtained from state laboratories (R. Timperi, personal communication) and could be corroborated with data from a screening study [26] for transfusion-acquired malaria in Canada.

Direct medical costs for individuals with NI and neurological sequelae from WNV were derived from published studies on other arboviral infections that lead to similar clinical outcomes. These studies included detailed estimates of resource utilization, including hospitalization, outpatient visits, and laboratory tests. Data from the US Bureau of Labor Statistics were used to assign a cost for the time required by an individual to care for a homebound patient on a full-time basis. To account for inflation, all costs were converted to 2003 US dollars by use of the Medical Care Component of the Consumer Price Index (http://www.bls.gov/).

## Results

### Face Validity of the Model

For the year 2002, the model predicts plausible numbers of transfusion-acquired WNV-associated NI cases. Assuming events are Poisson-distributed, these case predictions fall within the 95% tolerance intervals constructed by the observed numbers of transfusion-acquired WNV-associated NI cases by the relevant state health departments (Table 3).

**Table 3:**
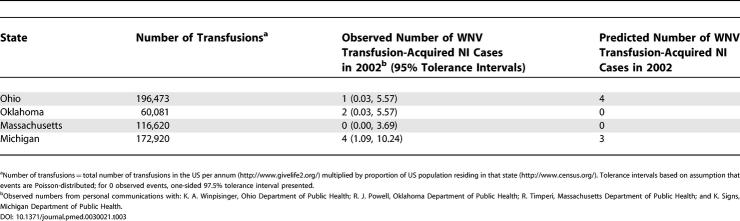
Observed and Predicted Number of Transfusion-Acquired WNV-Associated NI Cases in 2002 with Status-Quo Screening (Questionnaire)

### Projected Clinical and Economic Outcomes

The clinical outcomes of the different screening strategies in three different regions of varying transmission intensity are presented in Table 4. In the absence of specific screening for WNV, we projected, per 2 million transfusions, a total of 277 cases of WNV infection plus 50 cases of NI for the high-infection/short-duration epidemic scenario, and 205 cases of WNV infection plus 46 cases of NI for the high-infection/long-duration epidemic scenario. For the low-infection/short-duration epidemic scenario, we projected eight cases of WNV infection and one case of NI per 2 million transfusions.

**Table 4:**
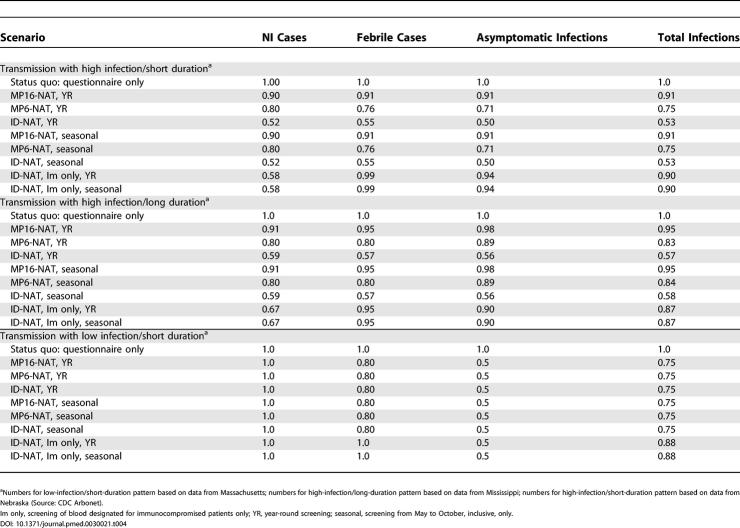
Predicted Clinical Outcomes Associated with Supplemental NAT Blood Screening Relative to Status-Quo Screening (Questionnaire)

Screening year-round did not prevent a greater number of cases than did seasonal screening from May to the end of October, even in areas with a long transmission season. The introduction of screening by ID-NAT had no impact in the low-intensity setting on the expected case numbers, but reduced the expected number of infections and cases of NI in the two higher-intensity settings. Screening with the MP6-NAT and MP16-NAT also reduced the number of infections and cases of NI, but was less effective than screening with ID-NAT for the higher-transmission-intensity settings.

While the strategy of restricting screening to blood designated for the immunocompromised population alone prevented fewer infections than screening the entire population of blood donors with ID-NAT, there was little impact on the number of cases of NI.

In the low-infection/short-duration scenario, the baseline strategy of screening through questionnaire alone was the least-costly and most cost-effective alternative; supplemental screening strategies increased the total cost significantly, but did not reduce the number of cases or increase quality-adjusted life expectancy in this setting (Table 5). In contrast, several of the screening strategies in the higher-intensity scenarios were less expensive and more cost-effective than the questionnaire alone, since the incremental costs of implementing these strategies were outweighed by the averted direct medical costs. In the high-intensity/short-duration setting, seasonal ID-NAT screening of blood designated for transfusion of immunocompromised patients was the most cost-effective of these strategies. Although seasonal screening of the entire donor pool with ID-NAT was nominally more effective, this was associated with an incremental quality-adjusted life expectancy benefit of less than 1 min. Comparable results were obtained for the high-intensity/long-duration scenario. In this setting again, seasonal screening with ID-NAT of blood designated for transfusion of immunocompromised patients dominated the other strategies, providing 3.6 min more of quality-adjusted life expectancy than the questionnaire alone and at less cost. Screening the entire donor pool with ID-NAT was slightly more effective at the cost of US$1.7 million/QALY gained.

**Table 5:**
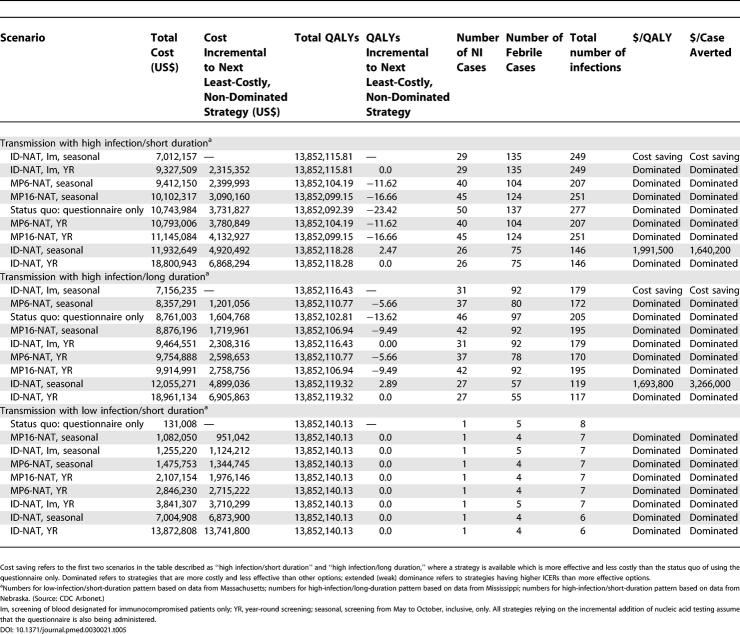
Predicted QALYs, Costs, Clinical Outcomes, and ICERs Associated with NAT Blood Screening for WNV in a Cohort of 2,000,000

### Sensitivity Analyses

Within areas of low infection /short duration of WNV, the questionnaire alone remained the least-costly strategy across a wide range of sensitivity analyses. When we assumed that assay sensitivity was as high as other nucleic acid tests, seasonally screening the entire donor pool by MP16-NAT was also on the efficiency curve; however, the ICER was US$1.2 million/QALY gained. Similarly, when we assumed an estimate from the high end of the plausible range for the probability of severe disease, ID-NAT added less than 1 min to the average quality-adjusted life expectancy at a cost of US$1 million/QALY. Further details are shown in Tables S1–S6.

For areas with high infection/short duration, the rank order of strategies was sensitive to variations in test sensitivity and the risk of developing severe disease. When we assumed the high assay sensitivity, seasonally screening the entire donor pool by MP6-NAT was the least-costly strategy; seasonally screening the entire donor pool by ID-NAT was also on the efficiency curve but exceeded US$7 million/QALY. When we assumed a high risk of severe disease, unrestricted seasonal screening by ID-NAT was the only non-dominated strategy. We also evaluated the effect of shortened seasonal screening from mid-July to mid-October versus full seasonal screening from May to the end of October for this transmission area. Shortened seasonal screening offered the same clinical benefit at lower cost than full seasonal screening; targeted screening of blood designated for transfusion of immunocompromised patients was the least-costly strategy. The ICER for universal screening by ID-NAT versus targeted screening of blood designated for transfusion of immunocompromised patients remained too high for universal screening to be a cost-effective strategy, even with shortened seasonal screening.

For the high-infection/long-duration scenario, our results were sensitive to both improved assay sensitivity and changes in assumptions about the risk of developing severe disease. When we assumed high assay sensitivity, seasonal screening by MP6-NAT was the only non-dominated strategy. When we assumed an estimate from the low end of the plausible range for risk of severe disease, the questionnaire strategy was least costly, although seasonal screening by ID-NAT of blood designated for transfusion of immunocompromised patients offered additional clinical benefit for an ICER of US$56,000/QALY gained.

## Discussion

The recent emergence of WNV in the US has led to a perceived need to safeguard the blood supply from viremic blood donations. Strategies for screening blood for emerging viral infections such as WNV are often put into place without systematic evaluation of their costs, benefits, and cost-effectiveness. In this study, we conducted a cost-effectiveness analysis of alternative strategies for blood screening and considered the efficacy of these strategies in areas with varying epidemic intensity, exploring the effect of variable assay characteristics, transfusion outcomes, and pricing that may affect current and future policy decisions.

Our analyses demonstrated that in areas with high infection rates, in the order of those seen in Mississippi and Nebraska in 2002, seasonal screening of blood designated for immunocompromised recipients prolongs quality-adjusted life expectancy compared with implementing a baseline questionnaire alone. Although other strategies, such as screening pooled samples designated for all donors, provided some benefit compared to a questionnaire alone, they were more costly and either less effective or only marginally more effective than restricted seasonal screening. In areas with low infection and seasonal transmission, none of the NAT strategies offered additional clinical benefit given current test-sensitivity estimates, although they were associated with substantial costs. These results suggest that the general screening of blood for WNV may not be as attractive a public health strategy as it first appeared to be, and that more restricted screening strategies may be preferable to currently mandated policies.

The finding that blood-screening strategies for WNV may be outside the usually accepted cost-effectiveness thresholds is consistent with previous cost-effectiveness analyses for blood screening for infectious agents [21,29–32]. A recent analysis of NAT screening for hepatitis B and C and HIV compared to serological testing alone showed that the ICER exceeded US$1.5 million/QALY gained, well beyond the US$50,000–100,000 threshold commonly used as an indicator of willingness to pay for a health-care intervention [21]. AuBuchon et al. [29] have previously enumerated some of the reasons why cost-effectiveness estimates of blood-screening tests are so unattractive; risks are relatively low, transfusion recipients often have a reduced quality-adjusted life expectancy, and costs are incurred for all donations, few of which are infectious. Despite this, blood-screening tests are often implemented for reasons that are not captured in a cost-effectiveness analysis. There is a perception that blood recipients cannot be held responsible for avoiding risk, and therefore the system must protect them at any cost. Individuals are willing to pay more to avoid a catastrophic outcome, even when the risk is low compared to other outcomes. Furthermore, policy makers are more likely to apply an intervention to a small and defined group such as blood recipients rather than to a less-visible group [32]. Nonetheless, in an era of major cuts in public health expenditure and increasingly limited resources available for health care, it is worthwhile reconsidering the economic implications of this priority; resources spent preventing the rare case of transfusion-associated WNV might be better utilized in a host of other interventions against infectious disease, including those focused on reducing WNV transmission through mosquito vectors. If such an approach were successful, it might obviate the need for screening blood for the virus in many areas.

Our analysis has a number of limitations as the ecology of WNV in the US, and the clinical course and sequelae of transfusion-acquired WNV infection, have not been clearly defined. Transmission intensities have varied over the years within some geographic regions since the emergence of WNV. Choosing the most cost-effective approach to screening within a specific area will depend on the ability to predict transmission intensity for the current season. Recently, a risk equation based on mosquito abundance, infectivity, vector competence, and host feeding behavior was developed to predict short-term future human WNV infections in an area [33]. The utility of this index to predict human infections is under investigation. Methods to validate, and improve upon, current prediction tools for WNV infection would enhance our ability to select the most cost-effective screening strategies. Improved methods to both measure and efficiently monitor the mosquito population parameters that determine virus transmission to humans would allow us to shift policy in response to important temporal changes in transmission patterns.

Lacking other data, we estimated the risk of developing NI after an infected transfusion from a single study in which patients with cancer were deliberately inoculated with WNV [13]. These data may overestimate the true risk of disease, since the patients studied may have been more susceptible to severe disease than healthier blood recipients. Such a bias would exaggerate the benefits of screening in our analyses. Otherwise, if the potentially higher dose of virus from a transfusion-associated infection results in an even higher risk of developing NI than we estimated, the benefits of screening are underestimated in our analyses [10,11]. In addition, in the absence of large-cohort data, we assumed that data from small studies on the long-term clinical consequences of WNV-associated NI patients represent the expected sequelae of infection.

Among the least well-defined parameters used in this analysis were those reflecting the performance of the newly introduced nucleic acid–screening tests. These were FDA-approved prior to establishing their sensitivity and specificity, and these characteristics have yet to be published. Given the imprecision of these estimates together with our expectation that the assay will improve with further product development, we repeated our analyses assuming that NAT for WNV was highly sensitive and specific. However, this analysis assumed that an improved test bore no additional cost. While various measures to enhance detection of low levels of viremia have been proposed, these would add further steps to screening rather than replace existing approaches, possibly adding substantially to expense. New methods that achieve small boosts in sensitivity (such as IgM antibody testing as an adjunct method to detect positive samples that have escaped NAT detection) are unlikely to be cost-effective under the assumptions made in this analysis. Custer et al. [34] demonstrated that while continuous ID-NAT screening would overburden blood-testing laboratories, ID-NAT screening during select times of the transmission season is needed currently, since minipool assays fail to detect 23% of the viremic samples detected by ID-NAT.

In conclusion, we found that NAT screening of blood donations for WNV improved clinical outcomes only in those areas where the incidence of WNV is high, and that limiting screening to high-intensity transmission seasons and to blood donations designated for immunocompromised patients reduced costs without decreasing quality-adjusted life expectancy in most scenarios. We recommend that states adopt screening policies based on the intensity and duration of their WNV epidemics. Regional data, in conjunction with the results of this analysis and consideration of societal risk attitudes and preferences, may collectively point to a relaxation of the current federally mandated NAT screening of all donations in low-intensity areas. When high rates of natural infection indicate that NAT screening is appropriate, we recommend use of ID-NAT rather than minipool screening. States should consider the restricted screening of blood designated for immunocompromised patients alone. Finally, we suggest that blood-screening policies be carefully scrutinized for cost-effectiveness and that their relative contribution to safeguarding public health be considered in the making of policy decisions.

## Supporting Information

Table S1 Predicted QALYs, Costs, Clinical Outcomes, and ICERs Associated with NAT Blood Screening for WNV in a Cohort of 2,000,000 High-sensitivity estimates.
(82 KB DOC).

Table S2 Predicted QALYs, Costs, Clinical Outcomes, and ICERs Associated with NAT Blood Screening for WNV in a Cohort of 2,000,000 Low-severity estimates.
(76 KB DOC).

Table S3 Predicted QALYs, Costs, Clinical Outcomes, and ICERs Associated with NAT Blood Screening for WNV in a Cohort of 2,000,000 High-severity estimates.
(76 KB DOC).

Table S4 Predicted QALYs, Costs, Clinical Outcomes, and ICERs Associated with NAT Blood Screening for WNV in a Cohort of 2,000,000 Low-cost estimates.
(76 KB DOC).

Table S5 Predicted QALYs, Costs, Clinical Outcomes, and ICERs Associated with NAT Blood Screening for WNV in a Cohort of 2,000,000 High-cost estimates.
(76 KB DOC).

Table S6 Predicted QALYs, Costs, Clinical Outcomes, and ICERs Associated with NAT Blood Screening for WNV in a Cohort of 2,000,000 Shortened seasonal screening for high-infection/short-duration transmission area.
(43 KB DOC).

**Patient Summary**

**Background**

West Nile virus (WNV) was first isolated from a sick woman in the West Nile region of Uganda in 1937. The virus has subsequently been found to be widespread in Africa and Eurasia, and sporadic outbreaks have been reported throughout these regions. WNV was first detected in the US in 1999, in a sick woman in New York. The disease has since spread to most states in the continental US, making thousands of people ill and causing several hundred deaths. Wild birds are the principal host of WNV, and the virus is transmitted to humans mainly by mosquitoes that bite both birds and humans. Most of the people who get infected by a mosquito bite do not get sick at all, but about 20% develop a flu-like illness. In a small number of cases—especially among the elderly and people with a weakened immune system—the infection spreads to the nervous system and can cause death or long-term disability. Like other blood-borne diseases, WNV can be transferred by blood transfusion with contaminated blood. Such cases have occurred in the US and have killed few people.

**Why Was this Study Done?**

WNV can be detected in blood samples by recently developed and approved tests. These tests detect most, but not all, cases of contamination with the virus. This means the WNV deaths resulting from transfusion of contaminated blood are potentially avoidable by screening donated blood. As a consequence, the US Food and Drug Administration (FDA) has mandated screening of donated blood samples. However, the FDA has not prescribed specific screening strategies, and the decision on how to best screen blood samples has been left to the individual states and the blood-collection agencies. The researchers who carried out this study wanted to determine which screening strategies would be cost-effective—that is, which strategies would prevent infections through contaminated blood for a reasonable price. In an ideal world, cost would not matter when it comes to protecting human life and health, but in reality there is limited money available for public health measures. Studies such as this one are therefore essential to help politicians decide how to spend the money.

**What Did the Researchers Do and Find?**

They calculated the costs of screening and the number of prevented infections through blood transfusion for a number of different scenarios. They found that in states with low WNV infection rates, the risk of an infected person donating blood was so low that screening was unlikely to prevent cases of serious illness from WNV, despite substantial costs. In states where WNV is common, screening throughout the year is likely to prevent cases of serious illness, but at a substantial cost. In states where WNV is common, screening blood only from May to the end of October (the months when mosquitoes are around and people get infected from them), however, was as effective at identifying contaminated blood samples as screening throughout the year. One way to reduce costs substantially was to create a separate blood pool that is reserved for transfusions to people with a weakened immune system and to screen only those samples. Because those are the people most at risk for severe WNV illness, this strategy would still prevent most of those cases.

**What Do These Findings Mean?**

It is not clear whether the current policy to screen all blood samples in all states makes sense from a health economy point of view. Restricting screening to states where WNV is common and to samples designated for people at higher risk for severe WNV illness would reduce costs significantly without putting the recipients of blood transfusions at a substantially higher risk of serious illness caused by WNV.

**Where Can I Get More Information Online?**

The following Web sites provide information on WNV.

Pages from the US Centers for Disease Control and Prevention:

http://www.cdc.gov/ncidod/dvbid/westnile/

Pages from the US National Biological Information Infrastructure:

http://westnilevirus.nbii.gov/

MedlinePlus pages:

http://www.nlm.nih.gov/medlineplus/westnilevirus.html

US Food and Drug Administration pages:

http://www.fda.gov/oc/opacom/hottopics/westnile.html

US Geological Survey pages:

http://westnilemaps.usgs.gov/

## Abbreviations

CDC: Centers for Disease Control and Prevention
FDA: Food and Drug Administration
ICER: incremental cost-effectiveness ratio
ID-NAT: individual nucleic acid test
MP6-NAT: nucleic acid testing of minipools of six samples
MP16-NAT: nucleic acid testing of minipools of 16 samples
NAT: nucleic acid test
NI: neuroinvasive disease
QALY: quality-adjusted life-year
WNV: West Nile virus
