# Supplementary material for: Cost-Effectiveness of Alternative Blood-Screening Strategies for West Nile Virus in the United States
Source: PLoS Med. 2006 Jan 24;3(2):e21. doi: 10.1371/journal.pmed.0030021 (PMC1324950; doi:10.1371/journal.pmed.0030021)
Supplement: Supplementary file 3 [file pmed.0030021.st003.doc]

**Table S3.** Predicted QALYs, Costs, Clinical Outcomes, and ICERs Associated with NAT Blood Screening for WNV in a Cohort of 2,000,000 (high severity estimates)

|  | Total Cost (US$) | Cost Incremental to Next Least-Costly, Non-Dominated Strategy (US$) | Total QALYs | QALYs Incremental to Next Least-Costly, Non-Dominated Strategy | Number of NI Cases | Number of Febrile Cases | | Total number of infections | | $/QALY | | $/Case Averted | |
| --- | --- | --- | --- | --- | --- | --- | --- | --- | --- | --- | --- | --- | --- |
| Transmission with high infection/short duration^a^ | | | | | | | | | | | | | |
| ID-NAT, seasonal | 22,102,988 | - | 13,852,070.96 | - | 73 | | 47 | | 146 | | Cost saving | | Cost saving |
| ID-NAT, Im, seasonal | 22,155,595 | 52,607 | 13,852,044.08 | -26.88 | 101 | | 93 | | 249 | | Dominated | | Dominated |
| MP6-NAT, seasonal | 22,423,460 | 320,472 | 13,852,043.90 | -27.06 | 97 | | 68 | | 207 | | Dominated | | Dominated |
| MP6-NAT, YR | 23,804,356 | 1,701,368 | 13,852,043.90 | -27.06 | 97 | | 68 | | 207 | | Dominated | | Dominated |
| ID-NAT, Im, YR | 24,470,947 | 2,367,959 | 13,852,044.08 | -26.88 | 101 | | 93 | | 249 | | Dominated | | Dominated |
| MP16-NAT, seasonal | 26,040,358 | 3,937,370 | 13,852,024.66 | -46.30 | 118 | | 81 | | 251 | | Dominated | | Dominated |
| MP16-NAT, YR | 27,083,125 | 4,980,137 | 13,852,024.66 | -46.30 | 118 | | 81 | | 251 | | Dominated | | Dominated |
| Status quo: questionnaire only | 27,439,776 | 5,336,788 | 13,852,014.45 | -56.51 | 128 | | 93 | | 277 | | Dominated | | Dominated |
| ID-NAT, YR | 28,971,282 | 6,868,294 | 13,852,070.96 | 0.0 | 73 | | 47 | | 146 | | Dominated | | Dominated |
| Transmission with high infection/long duration^a^ | | | | | | | | | | | | | |
| ID-NAT, Im, seasonal | 18,932,939 | - | 13,852,062.31 | - | 81 | | 58 | | 179 | | Cost saving | | Cost saving |
| MP6-NAT, seasonal | 20,371,335 | 1,438,396 | 13,852,056.27 | -6.04 | 85 | | 48 | | 172 | | Dominated | | Dominated |
| ID-NAT, seasonal | 20,700,818 | 1,767,879 | 13,852,079.89 | 17.58 | 60 | | 34 | | 119 | | 100,600 | | 84,200 |
| ID-NAT, Im, YR | 21,241,255 | 540,437 | 13,852,062.31 | -17.58 | 81 | | 58 | | 179 | | Dominated | | Dominated |
| MP6-NAT, YR | 21,280,839 | 580,021 | 13,852,058.46 | -21.43 | 84 | | 47 | | 170 | | Dominated | | Dominated |
| MP16-NAT, seasonal | 21,399,643 | 698,825 | 13,852,049.74 | -30.15 | 94 | | 58 | | 195 | | Dominated | | Dominated |
| Status quo: questionnaire only | 22,040,848 | 1,340,030 | 13,852,045.51 | -34.38 | 103 | | 59 | | 205 | | Dominated | | Dominated |
| MP16-NAT, YR | 22,438,438 | 1,737,620 | 13,852,049.74 | -30.15 | 94 | | 58 | | 195 | | Dominated | | Dominated |
| ID-NAT, YR | 27,130,525 | 6,429,707 | 13,852,082.03 | 2.14 | 59 | | 33 | | 117 | | 3,004,500 | | 6,429,700 |
| Transmission with low infection/short duration^a^ | | | | | | | | | | | | | |
| Status quo: questionnaire only | 770,208 | - | 13,852,137.27 | - | 5 | | 3 | | 8 | |  | |  |
| ID-NAT, Im, seasonal | 1,634,034 | 863,826 | 13,852,138.13 | 0.86 | 4 | | 3 | | 7 | | 1,004,400 | | 863,800 |
| MP16-NAT, seasonal | 1,721,250 | 87,216 | 13,852,137.27 | -0.86 | 5 | | 2 | | 7 | | Dominated | | Dominated |
| MP6-NAT, seasonal | 2,114,953 | 480,919 | 13,852,137.27 | -0.86 | 5 | | 2 | | 7 | | Dominated | | Dominated |
| MP16-NAT, YR | 2,746,354 | 1,112,320 | 13,852,137.27 | -0.86 | 5 | | 2 | | 7 | | Dominated | | Dominated |
| MP6-NAT, YR | 3,485,430 | 1,851,396 | 13,852,137.27 | -0.86 | 5 | | 2 | | 7 | | Dominated | | Dominated |
| ID-NAT, Im, YR | 4,220,121 | 2,586,087 | 13,852,138.13 | 0.0 | 4 | | 3 | | 7 | | Dominated | | Dominated |
| ID-NAT, seasonal | 7,383,722 | 5,749,688 | 13,852,138.13 | 0.0 | 4 | | 2 | | 6 | | Dominated | | Dominated |
| ID-NAT, YR | 14,251,632 | 12,617,598 | 13,852,138.13 | 0.0 | 4 | | 2 | | 6 | | Dominated | | Dominated |

Cost saving refers to the first two scenarios in the table described as “high infection/short duration” and “high infection/long duration”, where a strategy is available which is more effective and less costly than the status quo of using the questionnaire only. Dominated refers to strategies that are more costly and less effective than other options; extended (weak) dominance refers to strategies having higher ICERs than more effective options.

^a^Numbers for low-infection/short-duration pattern based on data from Massachusetts; numbers for high-infection/long-duration pattern based on data from Mississippi; numbers for high-infection/short-duration pattern based on data from Nebraska. (Source: CDC Arbonet.)

Im, screening of blood designated for immunocompromised patients only; YR, year-round screening; seasonal, screening from May to October, inclusive, only. All strategies relying on the incremental addition of nucleic acid testing assume that the questionnaire is also being administered.

DOI: 10.1371/journal.pmed.0030021.st003
